# A New and Unified Nomenclature for Male Fertility Restorer (RF) Proteins in Higher Plants

**DOI:** 10.1371/journal.pone.0015906

**Published:** 2010-12-28

**Authors:** Simeon O. Kotchoni, Jose C. Jimenez-Lopez, Emma W. Gachomo, Manfredo J. Seufferheld

**Affiliations:** 1 Department of Agronomy, Purdue University, Lilly Hall, West Lafayette, Indiana, United States of America; 2 Department of Biochemistry, Cell and Molecular Biology of Plants, Estacion Experimental del Zaidin (EEZ), Consejo Superior de Investigaciones Cientificas (CSIC), Granada, Spain; 3 Department of Botany and Plant Pathology, Purdue University, Lilly Hall, West Lafayette, Indiana, United States of America; 4 Department of Crop Science, University of Illinois U-C, Urbana-Champaign, Illinois, United States of America; National Institute for Medical Research, United Kingdom

## Abstract

The male fertility restorer (RF) proteins belong to extended protein families associated with the cytoplasmic male sterility in higher plants. Up till now, there is no devised nomenclature for naming the RF proteins. The systematic sequencing of new plant species in recent years has uncovered the existence of several novel *RF* genes and their encoded proteins. Their naming has been simply arbitrary and could not be adequately handled in the context of comparative functional genomics. We propose in this study a unified nomenclature for the RF extended protein families across all plant species. This new and unified nomenclature relies upon previously developed nomenclature for the first ever characterized *RF* gene, RF2A/ALDH2B2, a member of *ALDH* gene superfamily, and adheres to the guidelines issued by the ALDH Genome Nomenclature Committees. The proposed nomenclature reveals that *RF* gene superfamily encodes currently members of 51 families. This unified nomenclature accommodates functional *RF* genes and pseudogenes, and offers the flexibility needed to incorporate additional RFs as they become available in future. In addition, we provide a phylogenetic relationship between the RF extended families and use computational protein modeling to demonstrate the high divergence of RF functional specializations through specific structural features of selected members of RF superfamily.

## Introduction

Cytoplasmic male sterility (CMS) is a maternally inherited trait observed in numerous plant species, resulting in the formation of non-functional microspores or pollen grains [1], [2]. The most pronounced cytological events accompanying CMS concern the tapetum tissue surrounding the differentiating pollen mother cells (PMC), which involve its abnormal vacuolization, fusion of cells into multinuclear syncytia, and disturbances in the time of the programmed tapetum death [3], [4]. Development of PMC, is arrested either during meiosis or in postmeiotic phase, and is usually related to the failure in the deposition of the microspore (pollen) wall [1]. Mitochondrial function depends on the coordinate action of nuclear and mitochondrial genomes. CMS is generally determined by mitochondrial genomes. The regions whose expression is associated with CMS contain unusual ORFs that are often chimeric in structure and frequently co-transcribed with conventional mitochondrial genes [2].

In cells, nuclear genes called restorers of fertility (*RF*) have the ability to suppress the male-sterile phenotype and, hence, restore the production of pollen to plants carrying the deleterious mitochondrial genome. CMS/*RF* systems greatly facilitate hybrid seed production by eliminating the need for tedious hand emasculation and ensuring that each seed is a result of cross-pollination [5]. The *RF* allele from the pollen parent therefore restores fertility and seed production in the heterotic hybrid progeny. Apart from its commercial exploitation, CMS offers one of the few opportunities to examine the regulation of mitochondrial gene expression by a nuclear gene in multicellular organisms. Up to date, the mechanism by which CMS causes male sterility in higher plants is not fully known, and the functional features of male sterility restorer proteins, RFs, is completely unknown. In this study, we exclusively focused our attention on the RF extended gene families in higher plants. The study of nature and origin of genes that determine CMS, have provided new insights into plant mitochondrial-nuclear communication. This study has revealed the implication of mitochondrial signaling pathways, including those involved in regulating cell death and nuclear gene expression [6]. Generally, the nuclear RF genes encode pentatricopeptide-repeat (PPR) proteins as key regulators of plant mitochondrial gene expression [5]. However, in maize, the sterility restorer gene, *RF2*, which acts in conjunction with the *RF1* gene to restore fertility to T-cytoplasm maize, is an unusual restorer gene, and is the only one that has been well characterized [7]. Rather than affecting the expression of the CMS protein (URF13), the *RF2* is an aldehyde dehydrogenase [8] that acts by compensating for a metabolic defect caused by the low levels of URF13 protein. However, it is the presence of *RF1* that is responsible for reduction of the toxic protein (T-URF13) [9] and the alteration of the *T-URF13* transcript profile [10], [11]. In other words, the RF proteins are able to suppress mitochondrial abnormalities associated with male sterility. This suppression allows for normal metabolic processes leading to normal male reproductive organogenesis, successful microsporogenesis, pollen development and maturation. In many instances, the suppression is directly associated with *RF*-gene dependent mitochondrial RNA modification concurring in reduction of CMS-associated protein [5]. Different types of male sterility have been described [2], but the T-cytoplasm maize type (T-CMS) is the most intensively studied due to its role in the 1970 U.S. epidemic of southern corn leaf blight [12], [13]. At that time T-CMS was widely used in hybrid seed production because it eliminated the costly practice of hand detasseling. At the time of the epidemic ∼85% of the U.S. maize crop was produced using T-CMS, which is highly sensitive to host-selective toxins produced by both race T of *Cochliobolus heterostrophus* (asexual stage of *Bipolaris maydis*), the causal agent of southern corn leaf blight, and *Phyllosticta maydis,* the causal agent of yellow leaf blight [14].

Since the first *RF* was sequenced and identified in maize, the increasing numbers of *RF* genes have provided an ongoing challenge in their clear identification and logical classification across species. With the genome of more organisms being fully sequenced, significant information about newly sequenced eukaryotic genome content and organization has been revealed. The vast majority of higher plant genomes contain *RF* and *CMS* encoding genes. The recognition of the RF extended gene families has led us to a suggested revised nomenclature, which is based on the existing nomenclature of the first ever characterized RF gene from plants, RF2A/ALDH2B2 [15]–[19], and thereby conceptually close to *ALDH* gene superfamily nomenclature [15]–[18]. Previously, we have provided a revised/accepted nomenclature for the entire *ALDH* gene superfamily of rice [19] and maize including the maize RF2/ALD2B2 and its rice orthologs for the sake of unified standardization across all organisms [Kotchoni SO, unpublished]. In order to provide a unified nomenclature for the *RF* extended gene families, we have retrieved and characterized all *RF* cDNA/gene sequences from the GenBank/EMBL that have also been deposited as protein sequences in Swissprot/TrEMBL databases and uniformly named without discriminatory co-notation. In this nomenclature, all restorers of fertility were given “RF” as the route name for the protein superfamily, while the gene family and subfamily cataloguing were solely based on protein functionality and sequence similarity with previously characterized RF proteins. Similar to previously described ALDH protein superfamily nomenclature [15]–[19], protein sequences that are more than 40% identical to previously identified RF sequences compose a family, and sequences more than 60% identical within a family compose a protein subfamily. Subsequently, protein sequences that are less than 40% identical would describe a new RF protein family. This unified nomenclature offers the flexibility needed to incorporate additional RF proteins regardless of their origin, making comparative genomic studies between species very quick and easily understandable.

Despite the importance of RF proteins in the production of major crops such as rice and sunflower, as well as in the study of organelle-nuclear interactions in plants, to our knowledge, there is no systematic and comprehensive study of the entire members of RF protein superfamily across all taxa addressing their structural features and functional characterizations. In this study, we used a combination of functional genomics and computational biology to structurally and functionally characterize for the first time members of the restorer of fertility (RF) protein superfamily. Our data indicate that the RF protein superfamily consists of at least 51 divergent families, which will likely expand as more fully sequenced plant genomes become available.

## Results

### The restorer of fertility (RF) protein families: Revised and unified nomenclature

In order to provide a revised/international consensus and unified nomenclature for the *RF* gene superfamily, we first retrieved all the *RF* and *RF* like gene sequences using the molecular consensus patterns that define the fertility restoration related proteins such as the ALDH-GLU-active site (PS00687) and the ALDH-CYS-active site (PS00070), the pentatricopeptide (PPR) repeat profile (PS15375), the NB-ARC motif (PF00931), and the ATP/GTP-binding site motif A (PS00017), which are examples of the most representative consensuses. In an effort to highlight the entire molecular consensus characterizing the RF gene families, we provide in Table 1 the complete *RF*-defined molecular consensus patterns and their sequences used in this study for the validation of the retrieved gene sequences. We next verified all annotated plant RF open reading frames (ORFs) by comparing them to cDNA and EST sequences using sequence domains known to be homologous to well characterized RFs (Table 1). A complementary and comparative study was developed by using Uniprot database to validate the molecular function and previous denomination of each RF protein. Our searches resulted in the identification of 95 sequences that encode proteins with the diagnostic motifs described in Table 1 and Table S2. All the 95 full length sequences encode for RF and RF like proteins from a wide variety of plant species (Table 2, Table S2).

**Table 1:** Molecular consensus defining fertility the restorer protein structural patterns.

Molecular consensus	Name of sequence consensus	Code of consensus
[LIVMFGA] - E - [LIMSTAC] - [GS] - G - [KNLM] - [SADN] - [TAPFV]	ALDH_GLU_active site	PS00687
[FYLVA] - x - {GVEP} - {DILV} - G - [QE] - {LPYG} - C - [LIVMGSTANC] - [AGCN] - {HE} - [GSTADNEKR]	ALDH_CYS_active site	PS00070
	Pentatricopeptide (PPR) repeat profile	PS51375
Arg repeat regions	Arginine-rich region profile	PS50323
Pro repeat regions	Proline-rich region profile	PS50099
	NB-ARC	PF00931
[AG] - x(4) - G - K - [ST]	ATP/GTP-binding site motif A (P-loop)	PS00017
	TIR domain	PS50104
L - x(6) - L - x(6) - L - x(6) – L	Leucine zipper pattern	PS00029
Folylpolyglutamate synthase signature 1 [LIVMFY] - x - [LIVM] - [STAG] - G - T - [NK] - G - K - x - [STG] - x(4) - {A} - x - {EAD} - [LIVM](2) - x(3,4) - [GSKQT]	UDP-N-acetylmuramoyl-L-alanine:D-glutamate ligase (Mur ligase)	PS01011
Folylpolyglutamate synthase signature 2 [LIVMFY](2) - [EK] - x - G - [LIVM] - [GA] - G - x(2) - D - x - [GST] - x - [LIVM](2)	UDP-N-acetylmuramoyl-L-alanine:D-glutamate ligase (Mur ligase)	PS01012
[GSAT] - [KRHPSTQVME] - [LIVMFY] - x - [LIVMF] - [IVC] - [DN] - [LS] - [AH] - G - [SAN] – E	Kinesin motor domain	PS50067
[GSTALIVN] - {PCHR} - {KND} - H - E - [LIVMFYW] - {DEHRKP} - H - {EKPC} - [LIVMFYWGSPQ]	Neutral zinc metallopeptidases, zinc-binding region signature	PS00142
xxxxxxxxxxxxxxxxxx--------------xxxxxxxxxxxxxxxxx	Myc-type, “helix-loop-helix” domain profile	PS50888
Amphipathic helix 1 Loop Amphipathic helix 2		
C-X(8)-C-X(2)-CCysteine residues form three intramolecular disulfide bridges: C1-C5, C2-C3, and C4-C6	Ginkbilobin-2 (Gnk2)-homologous domain profile	PS51473
[STAGN] - {E} - [STAG] - [LIVMF] - R - L - {LP} - [SAGV] - N - [LIVMT]	ATP synthase a subunit	PS00449
[GSTA] - R - [NQ] - P - x(5) - {A} - x - {F} - x(2) - [LIVMFYW](2) - x(3) - [LIVMFYW] - x - [DE]	ATP synthase c subunit	PS00605
P - [SAP] - [LIV] - [DNH] - {LKGN} - {F} - {S} - S - {DCPH} - S	ATP synthase alpha/beta subunits	PS00152

**Table 2:** The fertility restorer protein superfamily: new and unified nomenclature.

RF Family	Revised annotation	Previous annotation	GeneBank acc. number	Protein Acc. number	Molecular pattern(s)	Putative functional characterization	Source
Family 1	RF1A1	RF2A	AF215823	Q43274	PS00687PS00070	ALDH (NAD^+^)	*Zea mays*
	RF1A2	RF2	AF269064	Q94G64	PS00070	ALDH	*Zea mays*
	RF1A3	RF2B	AF348418	Q7FWR0	PS00687PS00070	ALDH	*Zea mays*
	RF1A4	ALDH	AF162665	Q9LLR2	PS00687PS00070	ALDH	*Oryza sativa*
	RF1A5	RF2B	AF348417	Q8RUR9	PS00687PS00070	ALDH	*Zea mays*
	RF1B1	RF2C	BT063394	Q8S532	PS00687PS00070	ALDH	*Zea mays*
	RF1B2	RF2C	AF348413	Q8S531	PS00687PS00070	ALDH	*Zea mays*
	RF1B3	RF2D	BT041044	Q8S529	PS00687PS00070	ALDH	*Zea mays*
Family 2	RF2A1	RF1B	DQ311054	Q2PPE6	PS51375	PPR repeat	*Oryza sativa*
	RF2B1	RF1A	DQ311052	Q2PPE8	PS51375	PPR repeat	*Oryza sativa*
	RF2B2	RF1	AB106867	Q76C99	PS51375	PPR repeat	*Oryza sativa*
	RF2B3	RF1D	AB179840	Q6L6Q0	PS51375	PPR repeat	*Oryza sativa*
	RF2B4	PPR762	AB110443	Q76C24	PS51375	PPR repeat	*Oryza sativa*
	RF2B5	RFB	AB110443	Q769D0	PS51375	PPR repeat	*Oryza sativa*
	RF2B6	Os10g0497300	AP008216	Q76C22	PS51375	PPR repeat	*Oryza sativa*
	RF2B7	RF1C	AB112811	Q769C9	PS51375	PPR repeat	*Oryza sativa*
	RF2B8	PPR794	AB195686	Q76C26	PS51375	PPR repeat	*Oryza sativa*
	RF2C1	PPR-814a	FJ176574	C9W3P9	PS51375	PPR repeat	*Zea mays*
	RF2C2	PPR-814b	FJ184378	C9W4C1	PS51375	PPR repeat	*Zea mays*
	RF2C3	PPR-814c	FJ184379	C9W4C2	PS51375	PPR repeat	*Zea mays*
	RF2C4	PPR-817	FJ184376	C9W4B9	PS51375	PPR repeat	*Zea mays*
	RF2C5	PPR-816	FJ184377	C9W4C0	PS51375	PPR repeat	*Zea mays*
	RF2D1	AB110444	AB110444	Q76C21	PS51375	PPR repeat	*Oryza sativa*
	RF2D2	RF1B	AB112809	Q769D1	PS51375	PPR repeat	*Oryza sativa*
Family 3	RF3A1	RF1	AP008216	Q76C20	PS51375	PPR repeat	*Oryza sativa*
Family 4	RF4A1	Rf	DQ445625	Q84KB7	PS51375	PPR repeat	*Raphanus sativus*
	RF4A2	Ppr-B	EF472241	A4URR1	PS51375	PPR repeat	*Raphanus sativus*
	RF4A3	Rf	AB326285	A7BJL1	PS51375	PPR repeat	*Raphanus sativus*
	RF4A4	Rf	AB326284	A7BJL0	PS51375	PPR repeat	*Raphanus sativus*
	RF4A5	PPR	FJ593505	B9VQL7	PS51375	PPR repeat	*Raphanus sativus*
	RF4A6	PPR-A	AJ550021	C4WRH3	PS51375	PPR repeat	*Raphanus sativus*
	RF4A7	Ppr.24	AY285675	Q7XJ94	PS51375	PPR repeat	*Raphanus sativus*
	RF4A8	ppr-1	FN397617	D0R6K1	PS51375	PPR repeat	*Raphanus sativus*
	RF4A9	ppr-2	FN397617	D0R6K3	PS51375	PPR repeat	*Raphanus sativus*
	RF4A10	Ppr.27	AY285676	Q7X8E8	PS51375	PPR repeat	*Raphanus sativus*
	RF4A11	PPR-B-L1	FJ455099	B8XWY7	PS51375	PPR repeat	*Brassica napus*

In our previous report, we have provided a revised/unified nomenclature for the rice [19] and maize ALDH gene superfamily [Kotchoni SO, unpublished]. Adopting a standardized gene nomenclature, especially when there is no established naming consensus in the past is a very valuable contribution that can reduce and/or avoid data confusion in comparative genomic analysis, since the revised nomenclature is not based on source of the gene(s), but rather on sequence similarity to previously characterized members of the gene family (for detail, see materials and methods). Toward this goal, we systematically establish and provide the specific criteria for cataloguing/classifying the restorers of fertility (RF) in higher plants. The nomenclature is systematically structured to allow for the inclusion of newly identified or cloned RF genes and therefore genetically flexible for expansion. Our database search revealed a total of 95 *RF* genes encoding members of 51 *RF* gene families that are functionally characterized as restoring male sterility in higher plants (Table 2, Table S2). This unified nomenclature clarifies the nightmare and confusion of arbitrary gene annotation of the highly divergent *RF* genes characterized and deposited in gene bank databases so far and smartly eases data processes and classification in various comparative genomic studies and phylogenetic relationship between extended *RF* gene superfamily.

The new nomenclature criteria is structured with the high potential of expansion as more new genes will be cloned and deposited in gene bank databases. Interestingly, family 1 *RF* exclusively encodes for members of class 2 mitochondrial or cytosolic ALDHs (Table 2) and is evolutionarily distant from family 2 RF and the rest of the families. Currently, Family 2 *RF* is the most expended family with 16 gene members encoding for different multiple PPR repeat protein restorers followed by family 4 with 11 gene members encoding other PPR repeat RF proteins and by family 1 with 8 gene members encoding ALDH proteins that are highly divergent from the PPR repeat RF proteins (Table 2). The number of *RF* genes per species varies greatly from one plant species to another. Currently, *Oryza sativa* contains the highest number of *RF* genes followed by *Raphanus sativus* and *Zea mays* (Table 2, Table S2). At this time, more than half of the catalogued *RF* families encode members of single gene most of which represent the PPR repeat RF proteins and other less characterized functional domains (Table 1, Table 2, Table S2).

The total number of genes in the *RF* superfamily is expected to increase steadily with time, mainly due to the genomic sequencing of additional species. Regardless of the plethora of *RF* genes yet to be identified/characterized, their classification and relationship to the entire extended RF gene superfamily will be easy owing to this nomenclature building block that catalogues newly identified/characterized *RF* gene products only on the basis of sequence similarity to previously characterized *RF* gene products.

### Phylogenetic analysis of the extended RF protein families

**Figure 1:**
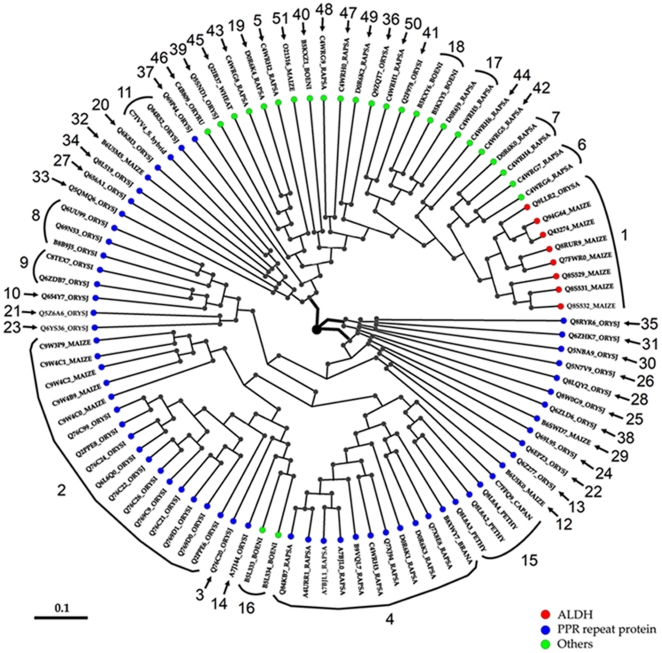
Phylogenetic analysis of plant fertility restorer proteins. Neighbor-Joining (NJ) method was used to perform a phylogenetic analysis of fertility restorer proteins from different families. The most abundant family members belong to the PPR repeat proteins (blue) followed by the ALDHs (red) and by the less abundant and divergent families (green). Plant species included in this analysis are maize, rice, Brassica, radish, wheat, Petunia, sugarcane, bell pepper and white ramie. Every new defined family of restorer protein has been depicted with the respective family number.

The retrieved full-length RF-related sequences were aligned to determine phylogenetic relationships within the male sterility restorer (RF) extended family. A phylogenetic tree of the RF extended sequences is depicted in Figure 1. The phylogenetic tree shows that the 51 RF extended families, although highly divergent, are split into three clades, with clades 1 and 2 representing mainly members of the PPR repeat RF proteins with the exception of family 16 RFs. Clade 3 represents uniquely members of ALDH proteins and the highly variable RF proteins distantly related to the PPR repeat proteins, but clustering together with the ALDH-RF proteins (Figure 1). The evolutionary relationships reveal some interesting observations. Family 1 exclusively represented by the ALDHs are male sterility restorers of monocots such as maize and rice, while the other RFs including the PPR repeat RF proteins are generally sterility restorers of other higher plant species (Figure 1). However, some members of family 2, 8–15, and family 32 PPR repeat RF proteins have also been identified in maize, rice (Table 2, Table S2).

### RF protein superfamilies: Structural and conformational variability

The crystallographic structural coordinates of relatively few RFs have been deposited in the Protein Database (PDB) so far. To our knowledge, detail comparative studies of structural and conformational features of members of the RF extended protein families have not been performed in higher plants. Using computational modeling analysis, we here determined for the first time the structural features and uniqueness of the 3D structures of selected members of the RF extended families. We wanted to appreciate in detail the structural divergence of the RFs mediating various functional specificities. Each sequence was modeled based on the ten best structural templates using the structural parameters summarized in Table 3.

**Table 3:** Structural-dependent modeling parameters for selected members of fertility restorer proteins.

Family	Accession number	Specie	Protein previous name	Template	Identity (%)
RF1A2	Q94G64	*Z. mays*	RF-2	1o01	63
RF12A1	B6U5K0	*Z. mays*	Fertility restorer	1w3bA	15
RF5A1	C4WRH2	*R. sativus*	AGD	1e8cB	28
RF49A1	D0R6K2	*R. sativus*	MOS-2	2ckkA	23
RF1A4	Q9LLR2	*O. sativa*	Aldehyde dehydrogenase	1ag8	61
RF14A1	A7J144	*O. sativa*	Fertility restorer	2q7fA	17

AGD: UDP-N-acetylmuramoylanalyl-D-glutamate-2-6-diaminoligase.

**Figure 2:**
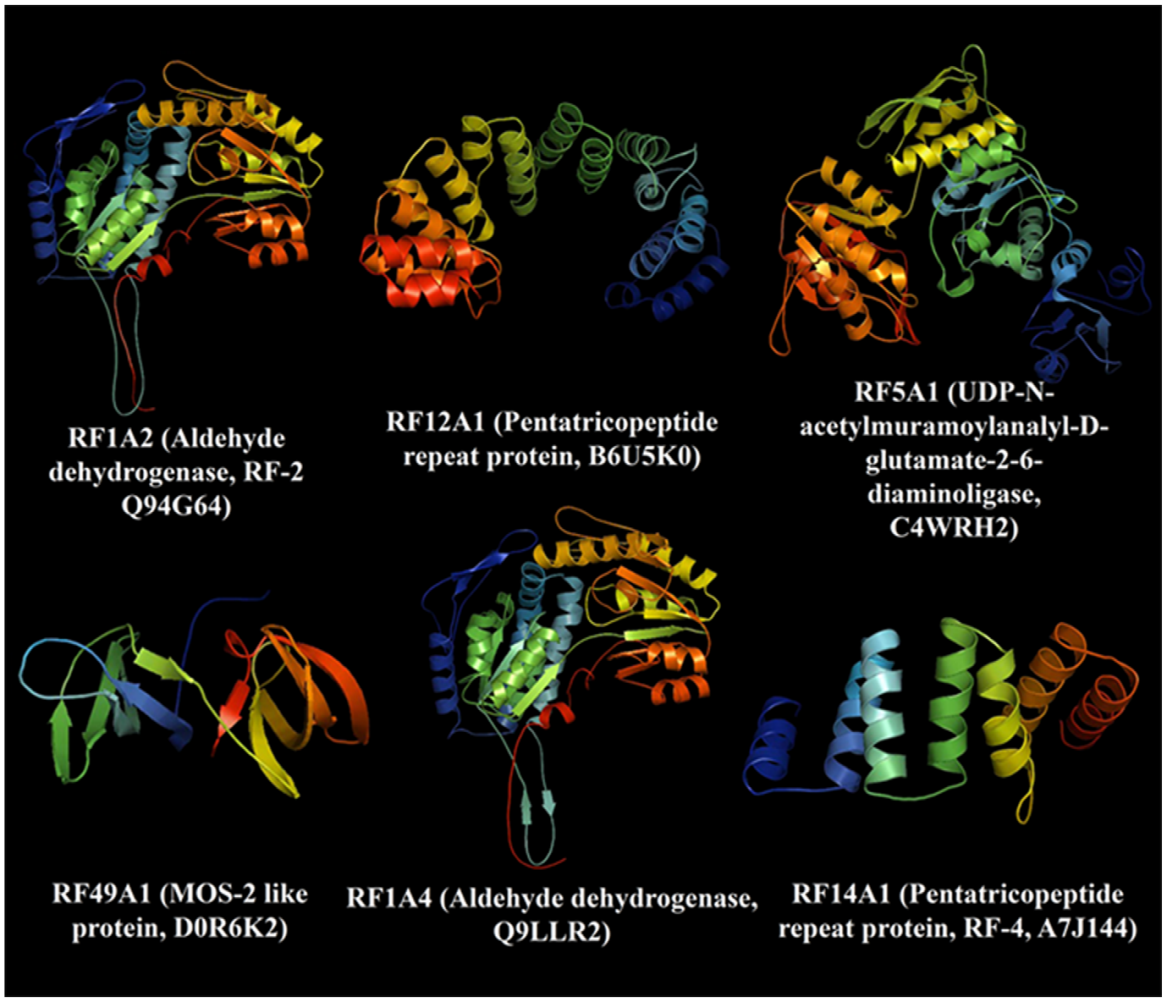
Three-dimensional structure analysis of selected members of cytoplasmic male sterility restorer proteins. The model proteins are depicted as cartoon diagrams. The secondary elements of the crystallographic structures are rainbow colored, with N-terminus in blue, and C-terminus in red.

**Figure 3:**
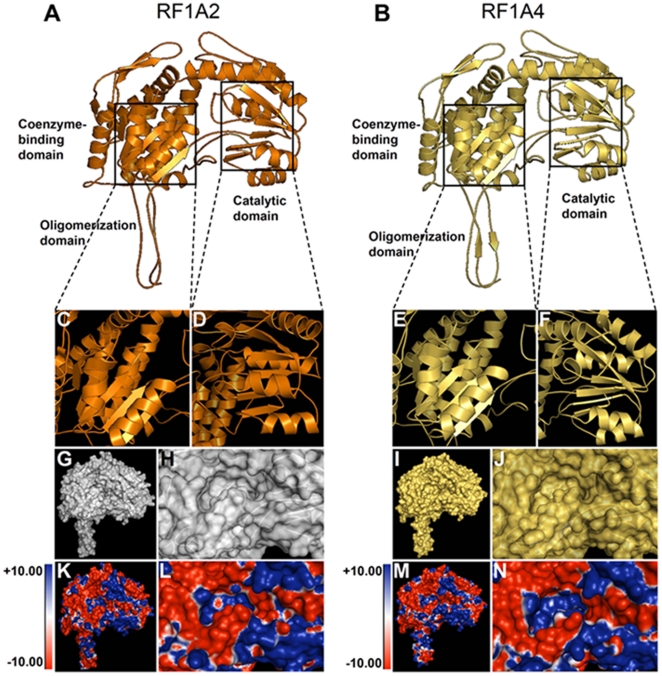
Rossmann-type fold of RF/ALDH proteins. (A, B) General structure (cartoon diagram) showing the secondary structural elements of RF1A2 (A) and RF1A4 (B). (C–F) Overview of the “Rossmann-type fold” structural features at coenzyme-binding domain and catalytic domain are depicted for RF1A2 (C,D) and RF1A4 (E, F). (G–J) Protein surface representation of RF1A2 (G) and RF1A4 (I). (H–J) Magnification overview of the coenzyme binding cavity of RF1A2 (H) and RF1A4 (J). (K–N) Electrostatic surface potential view of RF1A2 (K) and RF1A4 (M). Magnification overview of the electrostatic surface potential of the coenzyme binding cavity of RF1A2 (L) and RF1A4 (N). The surface colours are clamped at red (−10) or blue (+10).

A general structural comparison (Figure 2) and phylogenetic analysis (Figure 1) provided a clearer and unexpected insight into the structural divergence of the RF extended protein families. Our protein modeling data demonstrates the divergence of RF functional specializations highlighted here by very striking structural features of the selected members of RF extended protein families (Figure 2). The divergence in the molecular function is reflected by the differences in the structural subunit of the active RFs (Figure 2), i.e. each subunit of the dimeric or tetrameric enzyme ALDH of family 1 for instance. Each subunit of the active ALDHs is characterized by the “Rossmann fold”, and contains an NAD-binding domain, a catalytic domain and an oligomerization domain (Figure 2). At the interface of these domains there is a funnel-shaped opening leading to a putative catalytic pocket. In order to fully understand the structural characteristics of “Rossmann-type fold” of the RFs/ALDHs we depicted in Figure 3 the structural features of the RF/ALDH active site and the NAD-binding domain containing the Rassmann-type fold feature. The “Rossmann fold” represents the structural motif found in proteins that bind nucleotides, especially the NAD cofactor. The Rossmann fold structural feature is composed of three or more parallel beta strands linked by two alpha helices (Figure 3 C–F). Many members of the ALDH protein family possess different NAD-binding modes and catalytic sites, with a mechanism for enzymatic specificity and activity. Members of the pentatricopeptide repeat extended protein families are characterized by tandem repeats of a degenerate 35 amino acid motif that have a structure predicted to fold into a helix-turn-helix, similar to those found in previously characterized PPR proteins [20], and a degenerate 34 amino acid sequence in tandem arrays of 3–16 motifs, which form scaffolds to mediate protein-protein interactions (Figure 2).

## Discussion

Although many nuclear and mitochondrial genes associated with CMS have been characterized, the identification and characterization of *RF* genes has proven elusive, and only the maize *RF2A*, which encodes a class 2 mitochondrial ALDH is well characterized [2], [21]. However, orthologs of maize *FR2A* have been subsequently characterized in rice, and other plant species [19], [22].

*RF* is often associated with genes encoding pentatricopeptide repeat (PPR) proteins [23], [24]. PPR proteins constitute a large family, with more than 400 members in Arabidopsis, rice, maize, petunia, and Raphanus that are thought to be RNA binding proteins involved in posttranscriptional processes (RNA processing and translation) in mitochondria and chloroplasts [24]. Up to now, the RF gene extended families are deposited into the databases with arbitrary naming system by authors. This arbitrary nomenclature is not sustainable for adequate comparative mega-functional genomics studies, especially as the numbers of RF genes have increased steadily with the completion of more plant genome sequences. With the increase in genome sequencing of novel plant species, there are currently more than 800 genes encoding for RF or RF like proteins in plants. There are over 200 genes harboring the PPR-motif, and its related TPR (tetratricopeptide repeat)-motif in the *Arabidopsis* genome and two-thirds of these proteins are predicted to be targeted to organelles [24]. PPR- and TPR-motifs are found in helical-repeat proteins and would be predicted to have protein-binding properties. However, our data only revealed ∼100 RFs because we focused on RF genes that are indeed characterized as restorers of male sterility. These RF genes encode members of 51 gene families with Family 1 representing exclusively the aldehyde dehydrogenase cluster. The highly expanded PPR repeat RF genes encode for more than half (28 families) of the entire 51 family (Figure 1). With the exception of family 1, we know very little about the functions of members of other RF protein families. The PPR proteins have been hypothesized to function as sequence-specific adaptors for a variety of other RNA-associated proteins [25]. This was supported by the fact that the maize PPR protein CRP1 influences expression of chloroplast genes through association with specific mRNAs [26], and the fact that PPR proteins are involved in mRNA editing in chloroplasts [27]. In addition, the first RF gene identified in petunia, encoding the PPR protein Rf-PPR592, was suggested to have an mRNA processing function [5]. Our data reveals that about half of RF superfamily does not belong to the PPR repeat protein group (Table 2, Figure 2, Table S2). This new unified nomenclature provides essential inventories for comparative genomic analyses of the RF superfamily in flowering plants and the grass species. From the data, it appears that plant RFs have undergone functional specialization over time. Family 1 RF proteins are major fertility restorers of type T-cytoplasmic male sterility (T-CMS) in monocots, especially in maize and rice [28], while the cluster of PPR repeat protein families restore fertility to type S- and BT-CMS in various plant species [29], [30]. Several CMS/restorer systems are defined by the different origins of CMS with distinct genetic features. For instance, the BT-CMS (Boro II-type of CMS), WA-CMS (wild abortive-type of CMS) and HL-CMS (Honglian-type of CMS) are CMS types of rice, while the S-CMS (severely affected-type of CMS) and T-CMS (Texas-type of CMS) are of maize genotype, which arose spontaneously in a breeding line, and PET1-CMS of sunflower arose from an inter-specific cross between *Helianthus petiolaris* and *H. annuus*.

Our developed unified nomenclature system is helpful in a quick functional prediction of any newly cloned RF gene(s), because from the nomenclature point of view, the newly cloned gene(s) will always be characterized/named with sequence similarity with previously characterized RF genes/proteins. This modified and unifying nomenclature preserves also the widely arbitrary naming system used so far and referenced this old naming designation since the new name is linked to the gene accession number that will automatically pulled out the old naming system from the databases. The changes that have been introduced reflect into which extended family or subfamily a certain RF protein belongs. Accordingly, the new nomenclature will have no significant impact on already published data with old/arbitrary naming system. However, we urge scientists working on RFs to adopt this new and easy nomenclature system. In this regard, we have made an effort to preserve the user friendly linkage between the old and the new designations, which we hope will help researchers to adapt the new names. As the revised nomenclature should facilitate communication and understanding within the community interested in RFs, we advocate that this new naming system be used in all future studies.

## Materials and Methods

### Database search for RF genes

Restorer of fertility (RF) and RF-like gene sequences were retrieved from the US National Center for Biotechnology Information (NCBI, http://www.ncbi.nlm.nih.gov/) genome, the rice (TIGR Rice Annotation Release 4, http://tigrblast.tigr.org/eukblast/index.cgi?project=osa1), the maize (http://www.maizesequence.org) genome databases, and from the non-redundant expressed sequence tag (EST) databases using BLASTX, BLASTN and BLAST (low complexity filter, Blosum62 substitution matrix) [31]. The searches were conducted using previously characterized maize RF2A (GenBank Accession number AF215823), rice RF1A (GenBank Accession number DQ311052), rice RF1B (GenBank Accession number DQ311054), Brassica PPR-B-L1 (GenBank Accession number FJ455099) and Raphanus Mur-ligase (putative UDP-N-acetylmuranoylanalyl-D-2-6-diaminoligase) (GenBank Accession number AJ550021). Full-length amino acid sequences for fertility restorer proteins were compiled and aligned using ClustalW [32]. Genetic distances between pairs of amino acid sequences were calculated with Bioedit V7.0.5.3 [33]. Consensus protein sequences were derived from these original alignment, and further analyzed for the presence of putative functional motifs using the PROSITE database [34], [35], of biologically meaningful motif descriptors derived from multiple alignments and the ScanProsite program [36], from the Expert Protein Analysis System (ExPASy) proteomics server of the Swiss Institute of Bioinformatics [37]. Finally, the consensus protein sequences (Table 1) were submitted to BLASTP analysis to identify homologous proteins from other plant species. A comparative search for restorer protein homologous was performed using Uniprot database to confirm the identity of the retrieved RF proteins [38].

### Restorer of Fertility (RF) proteins: Revised/unified nomenclature

In order to provide a revised and unified nomenclature for *RF* gene superfamily, we developed a sequence based similarity approach to classify all the retrieved sequences using previously developed gene nomenclature model [15]–[19]. The criteria for cataloguing the RF protein superfamily was based on the established nomenclature criteria for cataloguing aldehyde dehydrogenase (*ALDH*) gene superfamily [15]–[19]; because ALDH (ALDH2B2/RF2A) being the first ever characterized plant RF gene, which was cloned from maize [7]. For this new nomenclature, RF protein sequences that are more than 40% identical to previously identified *RF* sequences compose a family, and sequences more than 60% identical within a family, compose a gene subfamily. Protein sequences that are less than 40% identical would describe a new *RF* gene family (Table S1). Taking maize *RF1A1* (previous name *FR2A*) as an example for the revised nomenclature, RF indicates the root; the first digit (1) indicates a family and the first letter (A) a subfamily, while the final number (1) identifies an individual gene within a subfamily. The revised nomenclature is therefore composed of an assigned gene *symbol* (*RF*) (abbreviated gene name) for the whole gene superfamily. The gene symbol must be (i) unique and representative of the gene superfamily; (ii) contain only Latin letters and/or Arabic numerals, (iii) not contain punctuation, and (iv) without any reference to species. These newly developed criteria have been applied to database curators to generate the unified *RF* gene families/classes regardless of the source of the cloned gene(s).

### Sequence alignments and phylogenetic analyses

The retrieved fertility restorer protein families were used to generate a phylogenetic tree using ClustalW [32]. The alignment was created using the Gonnet protein weight matrix, multiple alignment gap opening/extension penalties of 10/0.5 and pairwise gap opening/extension penalties of 10/0.1. These alignments were adjusted using Bioedit V7.0.5.3 [33]. Portions of sequences that could not be reliably aligned were eliminated. Phylogenetic tree was generated by the neighborjoining method (NJ), and the branches were tested with 1,000 bootstrap replicates. The three was visualized using Treedyn program [39].

### RF superfamily: protein modeling and structural characterization

In order to study the structural and conformational variability between the RF protein families, selected members of the RF superfamily were modeled using the best closed PDB templates structures using SWISS-MODEL, a protein structure homology-modeling server, via the ExPASy web server [40]–[42]. The initial modeled RF structures were subjected to energy minimization with GROMOS96 force field energy [43] implemented in DeepView/Swiss-PDBViewer v3.7 [44] to improve the van der Waals contacts and to correct the stereochemistry of the improved models. The quality of the models was assessed by checking the protein sterology with PROCHECK [45] and the protein energy with ANOLEA [46]. Ramachandran plot statistics for the models were calculated to show the number of protein residues in the favored regions.

## Supporting Information

Table S1 **Cross diagram representation of the sequence identity of the RF proteins.** The RF percentage identity to each other is used to the catalogue the RF families and subfamilies as detailed in materials and methods section.
(XLS)

Table S2 **The fertility restorer protein superfamily: new and unified nomenclature (continued).**
(DOC)
