# Supplementary material for: A New and Unified Nomenclature for Male Fertility Restorer (RF) Proteins in Higher Plants
Source: PLoS One. 2010 Dec 28;5(12):e15906. doi: 10.1371/journal.pone.0015906 (PMC3011004; doi:10.1371/journal.pone.0015906)
Supplement: Table S2 — The fertility restorer protein superfamily: new and unified nomenclature (continued). (DOC) [file pone.0015906.s002.doc]

Table S 2:

| RF Family | Revised annotation | Previous annotation | GeneBank acc. number | Protein Acc. number | Molecular pattern(s) | Putative functional characterization | Source |
| --- | --- | --- | --- | --- | --- | --- | --- |
| Family 5 | RF5A1 | Putative UDP-N-acetylmuranoylanalyl-D-2-6-diaminoligase | AJ550021 | C4WRH2 | PS01011  PS01012 | Mur Ligase | *Raphanus sativus* |
| Family 6 | RF6A1 | Putative uncharacterized protein | AJ550021 | C4WRG6 | PS51375 | PPR repeat | *Raphanus sativus* |
|  | RF6B1 | Putative uncharacterized protein | AJ550021 | C4WRG7 | PS51375 | PPR repeat | *Raphanus sativus* |
| Family 7 | RF7A1 | Putative uncharacterized protein | AJ550021 | C4WRH4 | PS51375 | PPR repeat | *Raphanus sativus* |
|  | RF7A2 | l7rfog3 | FN397617 | D0R6K0 | PS51375 | PPR repeat | *Oryza sativa* |
| Family 8 | RF8A1 | OSJNBa0024A05 | AY360390 | Q6UU99 | PS51375 | PPR repeat | *Oryza sativa* |
|  | RF8A2 | OSJNBa0061E21.110 | AP005693 | Q69N53 | PS51375 | PPR repeat | *Oryza sativa* |
|  | RF8A3 | K0486F02.25 | CM000133 | B8B9J5 | PS51375 | PPR repeat | *Oryza sativa* |
| Family 9 | RF9A1 | K0031E03.35 | AP009079 | C8TEX7 | PS51375 | PPR repeat | *Oryza sativa* |
|  | RF9A2 | P0045D08.129 | AY360394 | Q6ZDB7 | PS51375 | PPR repeat | *Oryza sativa* |
| Family 10 | RF10A1 | P0661G04.40 | [AP005457](http://www.ebi.ac.uk/ena/data/view/AP005457) | Q654Y7 | PS51375 | PPR repeat | *Oryza sativa* |
| Family 11 | RF11A1 | Os03g0736200 | DP000009 | Q84R52 | PS51375 | PPR repeat | *Oryza sativa* |
|  | RF11A2 | Sh265O22g_170 | AM403007 | C7IVV4 | PS51375 | PPR repeat | *Saccharum hybrid* |
| Family 12 | RF12A1 | Fertility restorer | EU972515 | B6U5K0 | PS51375 | PPR repeat | *Zea mays* |
| Family 13 | RF13A1 | OsJ_27230 | CM000145 | Q6Z277 | PS51375 | PPR repeat | *Oryza sativa* |
| Family 14 | RF14A1 | RF4 | DQ858155 | A7J144 | PS51375 | PPR repeat | *Oryza sativa* |
| Family 15 | RF15A1 | RF4 | [DQ858155](http://www.ebi.ac.uk/ena/data/view/DQ858155) | Q8L8A3 | PS51375 | PPR repeat | *Oryza sativa* |
|  | RF15A2 | rf-PPR592 | AY102721 | Q8L8A2 | PS51375 | PPR repeat | *Petunia hybrida* |
|  | RF15A3 | Rf-PPR592 | AY102719 | Q8L8A4 | PS51375 | PPR repeat | *Petunia hybrida* |
|  | RF15A4 | PPR1 | GQ365708 | C7FFQ9 | PS51375 | PPR repeat | *Capsicum annuum* |
| Family 16 | RF16A1 | atp9 | EU268006 | B5L333 | PS00605 | ATP synthase c subunit | *Boehmeria nivea* |
|  | RF16A2 | atp9 | EU268005 | B5L334 | PS00605 | ATP synthase c subunit | *Boehmeria nivea* |
| Family 17 | RF17A1 | Putative disease resistance protein | AJ550021 | C4WRH5 | PS50104  PF00931  PS00017  PS00029 | TIR domain;  NB-ARC;  ATP/GTP-binding site motif A (P-loop);  Leucine zipper pattern | *Raphanus sativus* |
|  | RF17A2 | Putative disease resistance protein | [FN397617](http://www.ebi.ac.uk/ena/data/view/FN397617) | D0R6J9 | PS50104  PF00931  PS00017  PS00029 | TIR domain;  NB-ARC;  ATP/GTP-binding site motif A (P-loop);  Leucine zipper pattern | *Raphanus sativus* |
| Family 18 | RF18A1 | atpA | EU122337 | B5KXY5 | PS00152 | ATP synthase alpha/beta | *Boehmeria nivea* |
|  | RF18A2 | atpA | EU122336 | B5KXY6 | PS00152 | ATP synthase alpha/beta | *Boehmeria nivea* |
| Family 19 | RF19A1 | l7rfog7 | FN397617 | D0R6K4 | PS01011  PS01012 | Mur Ligase | *Raphanus sativus* |
| Family 20 | RF20A1 | Os02g0468500 | [AP008208](http://www.ebi.ac.uk/ena/data/view/AP008208) | Q6K8I3 | PS51375 | PPR repeat | *Oryza sativa* |
| Family 21 | RF21A1 | P0513E02.9 | [AP005456](http://www.ebi.ac.uk/ena/data/view/AP005456) | Q5Z6A6 | PS51375 | PPR repeat | *Oryza sativa* |
| Family 22 | RF22A1 | Os09g0417500 | AP008215 | Q6EPZ3 | PS51375 | PPR repeat | *Oryza sativa* |
| Family 23 | RF23A1 | B1114D08.4 | AP006343 | Q6YS36 | PS51375 | PPR repeat | *Oryza sativa* |
| Family 24 | RF24A1 | B1026C12.18 | AP008213 | Q69L95 | PS51375 | PPR repeat | *Oryza sativa* |
| Family 25 | RF25A1 | P0529E05.16 | AP008207 | Q8W0G9 | PS51375 | PPR repeat | *Oryza sativa* |
| Family 26 | RF26A1 | P0470A12.47 | AK065343 | Q5N7V9 | PS51375 | PPR repeat | *Oryza sativa* |
| Family 27 | RF27A1 | P0596H06.19 | AP008212 | Q656A1 | PS51375 | PPR repeat | *Oryza sativa* |
| Family 28 | RF28A1 | P0046B10.15 | AP008207 | Q8LQY2 | PS51375 | PPR repeat | *Oryza sativa* |
| Family 29 | RF29A1 | Fertility restorer | EU957052 | B6SWD7 | PS51375 | PPR repeat | *Zea mays* |
| Family 30 | RF30A1 | P0453A06.6 | AP001383 | Q5NBA9 | PS51375 | PPR repeat | *Oryza sativa* |
| Family 31 | RF31A1 | OJ1218_C12.18 | AP004051 | Q6ZHK7 | PS51375 | PPR repeat | *Oryza sativa* |
| Family 32 | RF32A1 | RF1 | EU972540 | B6U5M5 | PS51375 | PPR repeat | *Zea mays* |
| Family 33 | RF33A1 | P0007F06.6 | AP003706 | Q5QMQ6 | PS51375 | PPR repeat | *Oryza sativa* |
| Family 34 | RF34A1 | P0497A05.20 | AP003416 | Q8L519 | PS51375 | PPR repeat | *Oryza sativa* |
| Family 35 | RF35A1 | OSJNBa0026J14.19 | AP008207 | Q8RYR6 | PS51375 | PPR repeat | *Oryza sativa* |
| Family 36 | RF36A1 | DD2-3 | AB017914 | Q9ZQT7 | PF00931  PS00017  PS00029 | NB-ARC;  ATP/GTP-binding site motif A (P-loop);  Leucine zipper pattern | *Oryza sativa* |
| Family 37 | RF37A1 | OJ1740_D06.41 | [AP005579](http://www.ebi.ac.uk/ena/data/view/AP005579) | Q69P44 | PS51375 | PPR repeat | *Oryza sativa* |
| Family 38 | RF38A1 | OJ1014_E09.3 | AP003826 | Q6ZLD6 | PS51375 | PPR repeat | *Oryza sativa* |
| Family 39 | RF39A1 | OSJNBa0014B15.9 | AP002854 | Q5SND1 | PS50323  PS50099 | Arginine-rich region profile;  Proline-rich region profile | *Oryza sativa* |
| Family 40 | RF40A1 | atp6 | EU122342 | B5KXZ1 | PS00449 | ATP synthase α subunit | *Boehmeria nivea* |
| Family 41 | RF41A1 | orf161 | AP011077 | Q2F978 | - | Non-known region function | *Oryza sativa* |
| Family 42 | RF42A1 | CAZ40327.1 | AJ550021 | C4WRG5 | - | Non-known region function | *Raphanus sativus* |
| Family 43 | RF43A1 | CAZ40330.1 | AJ550021 | C4WRG8 | PS51473 | Ginkbilobin-2 (Gnk2)-homologous domain profile | *Raphanus sativus* |
| Family 44 | RF44A1 | Putative aminopeptidase | AJ550021 | C4WRH6 | PS00142 | Neutral zinc metallopeptidases; zinc-binding region signature | *Raphanus sativus* |
| Family 45 | RF45A1 | fertility relative protein RF1 | DQ017898 | Q2IB37 | PS00029 | Leucine zipper | *Triticum aestivum* |
| Family 46 | RF46A1 | Rf17 | AB481199 | C4B809 | - | Non-known region function | *Oryza rufipogon* |
| Family 47 | RF47A1 | Putative transcription factor | AJ550021 | C4WRH0 | PS50888 | Myc-type, “helix-loop-helix” domain profile | *Raphanus sativus* |
| Family 48 | RF48A1 | Putative kinesin-like protein | AJ550021 | C4WRG9 | PS50067 | Kinesin motor domain | *Raphanus sativus* |
| Family 49 | RF49A1 | l7rfog5 | FN397617 | D0R6K2 | Non-classified | Putative MOS-2 like protein (Innate Immunity in Plants) | *Raphanus sativus* |
| Family 50 | RF50A1 | CAZ40333.1 | AJ550021 | C4WRH1 | - | Non-known region function | *Raphanus sativus* |
| Family 51 | RF51A1 | ORF355 | AJ550021 | O21316 | - | Non-known region function | *Zea mays* |
